# Beyond Newborn Screening: Clinical Outcomes and Genotypic Spectrum Underlying Maple Syrup Urine Disease in Mexico

**DOI:** 10.3390/ijns12040083

**Published:** 2026-10-09

**Authors:** Marcela Vela-Amieva, Miriam Erandi Reyna-Fabián, Liliana Fernández-Hernández, Sara Guillén-López, Lizbeth López-Mejía, Miguel Angel Alcantara-Ortigoza, Ariadna Gonzalez-del Angel, Bernardette Estandía-Ortega, Isabel Ibarra-González, Laura Paz-Castillo, Cynthia Fernández-Lainez

**Affiliations:** 1 Laboratorio de Errores Innatos del Metabolismo y Tamiz, Instituto Nacional de Pediatría, Secretaría de Salud, Mexico City 04530, Mexico; mvelaa@pediatria.gob.mx (M.V.-A.);; 2 Laboratorio de Biología Molecular, Instituto Nacional de Pediatría, Secretaría de Salud, Mexico City 04530, Mexico; 3 Unidad de Genética de la Nutrición, Instituto de Investigaciones Biomédicas, National Autonomous University of Mexico (UNAM), Mexico City 04530, Mexico; 4 Servicio de Endocrinología, Hospital de la Niñez Oaxaqueña, Oaxaca 71294, Mexico

**Keywords:** newborn screening, public health, post-screening, short and long-term follow-up

## Abstract

Maple syrup urine disease (MSUD) is a time-critical inborn error of metabolism in which early detection may prevent irreversible neurological injury, yet the success of newborn screening (NBS) depends on timely post-screening care. We retrospectively characterized the clinical, biochemical, and genotypic spectrum of 88 Mexican patients from 80 unrelated families evaluated between 1995 and 2026 and assessed the relationship between NBS, treatment timing, neurological outcome, and survival. Most patients had severe neonatal disease, and 73/88 (83%) presented with acute metabolic decompensation requiring hospitalization. Nine patients were identified by NBS: six received timely confirmatory diagnosis and treatment and maintained normal age-appropriate neurodevelopment during 10 years of follow-up, whereas three experienced delays in the diagnostic and therapeutic pathway, developed metabolic decompensation, and subsequently had severe neurological disability. None of the successfully screened patients died. Among 33 molecularly characterized unrelated patients, 31 distinct variants were identified in *BCKDHA*, *BCKDHB*, and *DBT*, with marked genotypic heterogeneity. These findings show that National NBS strategies should prioritize the inclusion of this time critical disorder and the shortening of the interval between a positive screening result and treatment initiation. MSUD NBS should serve as the entry point to an optimal continuum of care. Rapid confirmation, immediate referral, treatment initiation, and sustained specialized metabolic follow-up are essential for translating early detection into favorable outcomes.

## 1. Introduction

**Figure 1:**
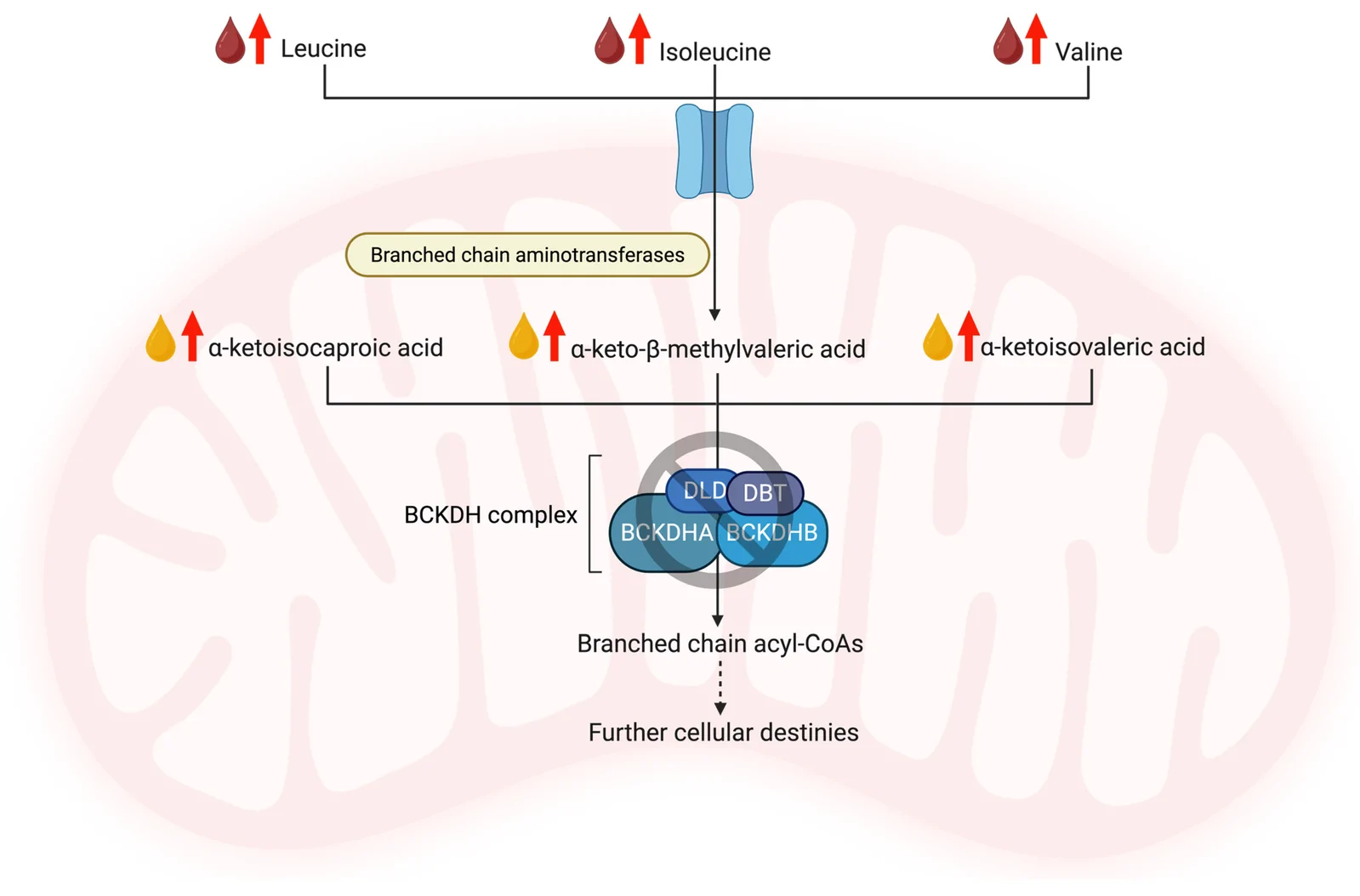
Biochemical pathway of branched-chain amino acids and the enzymatic blockade causative of MSUD. Abbreviations: BCKDH, branched-chain α-keto-acid dehydrogenase; BCKDHA, branched-chain α-keto-acid dehydrogenase subunit α (E1α); BCKDHB, branched-chain α-keto-acid dehydrogenase subunit β (E1β); DLD, dihydrolipoamide dehydrogenase (E2); DBT, dihydrolipoamide branched-chain transacylase (E3). Red drop indicates blood; yellow drop indicates urinary excretion. Red arrows indicate blood or urine elevated concentration.

Maple syrup urine disease (MSUD; OMIM #248600) is a rare autosomal recessive inborn error of metabolism caused by deficiency of the mitochondrial branched-chain α-ketoacid dehydrogenase (BCKDH) complex, which catalyzes the second step in the catabolism of the branched-chain amino acids (BCAAs) leucine, isoleucine, and valine [1]. Deficient BCKDH activity leads to the accumulation of BCAAs and their corresponding branched-chain α-ketoacids in blood and tissues, leading to metabolic intoxication, cerebral edema, and progressive neurological injury (Figure 1).

Among the accumulated metabolites, leucine is considered the primary neurotoxic compound and is strongly associated with acute encephalopathy, seizures, and long-term neurodevelopmental impairment [2].

MSUD was first described in 1954 by Menkes and colleagues and has an estimated worldwide incidence of approximately 1 in 185,000–200,000 live births. However, substantially higher frequencies have been reported in founder populations such as the Old Order Mennonites of Pennsylvania and certain Portuguese and Asian populations [1,3,4,5].

Clinically, MSUD encompasses a broad phenotypic spectrum ranging from a severe classic neonatal form to intermediate, intermittent, and thiamine-responsive variants, reflecting substantial biochemical and clinical heterogeneity [1].

In patients with classic MSUD, symptoms typically develop within the first days of life and include poor feeding, lethargy, irritability, abnormal muscle tone, and rapidly progressive encephalopathy. Without prompt diagnosis and treatment, affected neonates may develop cerebral edema, coma, and death within the first weeks of life [1,6].

Because irreversible neurological injury may occur within the first days of life, MSUD is considered one of the most important time-critical disorders included in newborn screening (NBS) programs [7]. Tandem mass spectrometry-based NBS enables presymptomatic detection by measuring elevated leucine/isoleucine concentrations in dried blood spots and has substantially improved survival and neurodevelopmental outcomes in affected infants [8,9].

However, increasing evidence indicates that early detection alone does not guarantee favorable outcomes. Several studies have demonstrated that the success of NBS depends not only on analytical performance but also on the efficiency of the entire continuum of care, including rapid confirmatory diagnosis, immediate referral to specialized metabolic centers, timely initiation of treatment, access to specialized medical formulas, and appropriate management of metabolic crisis [9,10]. Furthermore, some neonates with severe classic MSUD may develop metabolic intoxication before NBS results become available, limiting the ability of NBS alone to prevent neurological injury [11,12]. These observations highlight that, for time-critical disorders such as MSUD, patient outcomes depend not only on early detection but also on the quality of post-screening metabolic care.

In Mexico, the Ministry of Health’s mandatory NBS program includes only six diseases: congenital hypothyroidism, phenylketonuria, congenital adrenal hyperplasia, galactosemia, cystic fibrosis, and glucose-6-phosphate dehydrogenase deficiency [13]. Expanded screening for MSUD using tandem mass spectrometry is performed as a complementary test at other institutions within the National Health System and in the private sector. Consequently, access to MSUD screening remains heterogeneous and depends largely on the healthcare subsystem in which the newborn receives medical care.

The BCKDH complex is composed of four catalytic components encoded by the *BCKDHA* (19q13.2, MIM*608348; MSUD Type Ia, MIM#248600), *BCKDHB* (6q14.1, MIM*248611; MSUD Type Ib, MIM#620698), *DBT* (1q21.2, MIM*248610; MSUD Type II, MIM#620699), and *DLD* (7q31.1, MIM*238331; Dihydrolipoamide dehydrogenase deficiency, MIM#246900) genes. Biallelic pathogenic variants in any of these genes can cause MSUD by reducing BCKDH enzyme activity to varying degrees [1,14]. More than 250 different disease-causing variants have been reported worldwide, including missense, nonsense, splice-site, frameshift, and structural variants, according to the Leiden Open Variation Database (LOVD). While founder variants have been identified in isolated populations, most countries exhibit extensive allelic heterogeneity and a predominance of private or family-specific variants [1,15]. This molecular diversity complicates genotype–phenotype correlations and underscores the importance of population-specific studies to improve molecular diagnosis, genetic counseling, and the interpretation of disease-associated variants.

In Latin America, knowledge of the molecular epidemiology of MSUD remains limited and is largely derived from relatively small cohorts. Studies from Chile have identified both recurrent and novel pathogenic variants, with a predominance of *BCKDHB* variants and limited evidence of consistent genotype–phenotype correlations [15]. Likewise, recent studies from Brazil have demonstrated extensive allelic heterogeneity and the absence of major founder variants, suggesting a highly diverse genotypic landscape across the region [16]. Although several countries have incorporated MSUD into their NBS programs, data regarding the impact of screening on long-term clinical outcomes remains scarce.

In Mexico, available information on MSUD is limited to isolated case reports and small clinical series, and its genotypic spectrum has not been systematically characterized [17,18]. Likewise, the success of NBS and the factors influencing neurological outcomes in Mexican patients have not been comprehensively evaluated. This knowledge gap limits the development of evidence-based diagnostic, therapeutic, and public health strategies.

Therefore, the aim of the present study was to characterize the clinical, biochemical, and genotypic spectrum of MSUD in the largest Mexican cohort reported to date and to evaluate the impact of NBS on clinical outcomes. Specifically, we sought to identify genotypic and NBS-related factors associated with neurological disability and mortality in the Mexican MSUD patients. By integrating genotypic findings with long-term clinical follow-up, this study provides insights into the challenges that remain beyond NBS and highlights the healthcare factors that continue to influence outcomes in patients with MSUD.

## 2. Materials and Methods

### 2.1. Study Design and Setting

A retrospective observational cohort study was conducted at the National Institute of Pediatrics (Instituto Nacional de Pediatría, INP), a national tertiary referral center for inherited metabolic disorders in Mexico. Clinical, biochemical, molecular, treatment, and follow-up data from patients evaluated between January 1995 and May 2026 were retrospectively reviewed.

### 2.2. Study Population

Mexican patients with a confirmed biochemical diagnosis of MSUD enrolled in the institutional Inborn Errors of Metabolism cohort were eligible for inclusion. Patients diagnosed either through newborn screening or after clinical presentation were included, provided they met the predefined biochemical diagnostic criteria.

Biochemical diagnosis was established using previously validated and published protocols [19,20] including: (1) Elevated blood concentrations of leucine/isoleucine and valine detected by tandem mass spectrometry (MS/MS); (2) Increased plasma concentrations of leucine, valine, and alloisoleucine measured by high-performance liquid chromatography (HPLC); and (3) Detection of characteristic branched-chain ketoacids, including α-ketoisocaproic acid, α-ketoisovaleric acid, 2-keto-3-methylvaleric acid, and 2-hydroxyisovaleric acid, by gas chromatography coupled with mass spectrometry (GC-MS).

### 2.3. Clinical and Biochemical Data Collection

Clinical data were obtained from medical records, the institutional metabolic clinic database, and institutional laboratory databases. The following variables were collected: sex, family history of MSUD, family history of deceased siblings with an unknown diagnosis, consanguinity, age at symptom onset, age at diagnosis, age at treatment initiation, NBS status, clinical presentation at diagnosis, number of metabolic decompensations requiring hospitalization, neurological outcome, and survival status at the last follow-up. Clinical manifestations were standardized according to the Human Phenotype Ontology (HPO) terminology [21].

To assess neurological outcome in patients under 6 years old, neurodevelopmental delay was assessed using the Denver Developmental Screening Test [22]. For older patients with intellectual disability, the Patel classification was used [23].

Biochemical variables included plasma concentrations of leucine, isoleucine, valine, and alloisoleucine at diagnosis. Because alloisoleucine quantification was incorporated into the institutional diagnosis protocol during the study period, this variable was available only for patients evaluated after its implementation.

### 2.4. Phenotypic Classification

Patients were classified according to established clinical criteria into classic, intermediate, intermittent, and thiamine-responsive MSUD phenotypes. The classic phenotype was defined by neonatal-onset disease with severe encephalopathy, feeding difficulties, lethargy, and marked biochemical abnormalities during the first days of life. The intermediate phenotype was considered by a variable age of onset, and their phenotype was similar to the classic form, but less severe, with a milder clinical course and later or less severe manifestations. Even though there are no absolute phenotypic distinctions in absence of enzymatic activity data. Intermittent phenotype was defined as normal growth and normal neurodevelopment between episodes of metabolic decompensation. Thiamine-responsive MSUD was defined as those patients with demonstrated clinical and biochemical improvement after thiamine supplementation at a dose of 100–200 mg/day [1,24].

### 2.5. Molecular Genetic Analysis

Genomic DNA was isolated from dried blood samples (DBS) using standard methods with the QIAamp^®^ DNA micro kit (QIAGEN^®^, Hilden, Germany) according to the manufacturer’s instructions.

Molecular analysis was performed for all patients included in the study using next-generation sequencing (NGS)-based approaches, depending on the period in which each patient was evaluated and the diagnostic technology available at that time. Depending on clinical availability, patients underwent clinical exome sequencing, whole-exome sequencing (WES), or phenotype-driven targeted gene panel analysis, all performed by accredited private clinical diagnostic laboratories. Sequencing libraries were prepared following the manufacturers’ protocols, and captured libraries were sequenced on Illumina platforms (Illumina^®^, San Diego, CA, USA). Sequence reads were aligned to the human reference genome (GRCh37/hg19), and variants were identified using validated bioinformatic pipelines.

Variants were described according to the Human Genome Variation Society (HGVS) nomenclature using the corresponding RefSeq transcript for each gene and were classified following the recommendations of the American College of Medical Genetics and Genomics and the Association for Molecular Pathology (ACMG/AMP). Only variants considered clinically relevant to the patient’s phenotype were included in the analysis. When clinically indicated, selected variants were confirmed by Sanger sequencing, and segregation analysis was performed in available family members.

### 2.6. Metabolic and Nutritional Management

Nutritional management was provided according to the Nutritional Management Guideline for Individuals with MSUD developed by the Southeast Regional Genetics Network (SERN) and Genetic Metabolic Dietitians International (GMDI) [24].

Treatment consisted of individualized dietary restriction of BCAAs through controlled natural protein intake, combined with supplementation with a BCAA-free medical formula to meet age-specific protein requirements. Dietary prescriptions were adjusted according to age, nutritional status, growth, and biochemical monitoring. Plasma amino acids concentrations were assessed periodically to optimize metabolic control. Supplementation with valine and isoleucine was prescribed when indicated to maintain physiological BCAA balance and prevent deficiencies.

During episodes of metabolic decompensation, emergency metabolic management protocols were implemented to reduce catabolism and prevent neurological injury. None of the patients met the criteria for thiamine-responsive MSUD and therefore did not receive thiamine as monotherapy. However, all patients received adjunctive thiamine supplementation (100–200 mg/day) in combination with nutritional treatment, consistent with current clinical practice guidelines (SERN/GMDI) [24].

### 2.7. Outcome Measures

The primary outcomes were neurological outcomes at the last follow-up and survival status. Additional variables included age at diagnosis, biochemical profile at diagnosis, MSUD genotype, clinical phenotype, and NBS status.

The genotypic analysis included the types and distribution of pathogenic/likely pathogenic variants across the *BCKDHA*, *BCKDHB*, *DBT*, and *DLD* genes, as well as the evaluation of genotype–phenotype correlations. Clinical outcomes were also compared between patients identified through NBS and those diagnosed after clinical presentation.

### 2.8. Evaluation of Newborn Screening Outcomes

For patients identified through NBS, the intervals between sample collection, availability of the screening result, confirmatory diagnosis, and treatment initiation were recorded. Factors contributing to delayed or disrupted screening pathway which avoided the prevention of neurological injury were identified through a retrospective review of complete clinical records, using predefined operational criteria [25,26,27]. These factors were classified as: (1) analytical factors, including limitations of the screening methodology and false-negative results; (2) post-analytical factors, including delays in referral, confirmatory diagnosis, treatment initiation, or clinical management; and (3) healthcare access factors, including limited availability of specialized metabolic care or medical formulas.

### 2.9. Mutational Spectrum and Genotype–Phenotype Analysis

Patients were first classified according to the affected gene (*BCKDHA*, *BCKDHB*, *DBT*, or *DLD*). For exploratory genotype–phenotype analyses, variants were further categorized according to their predicted functional consequences as: (1) predicted loss-of-function (pLOF) variants, including nonsense, frameshift, and canonical splice-site variants); (2) predicted non-loss-of-function (pnLOF) variants, including missense variants, and (3) variants of uncertain significance (VUS) [28].

Associations between genotype and clinical phenotype were explored by comparing the frequencies of neonatal presentation, severe metabolic decompensation, neurological impairment, and mortality across molecular groups.

### 2.10. Patient Follow-Up

Follow-up duration was calculated from the date of diagnosis to the date of the last documented clinical evaluation, death, or loss of follow-up, whichever occurred first. At the end of the study period, patients were classified as alive, deceased, or loss of follow-up.

### 2.11. Missing Data

Given the retrospective nature of the study, missing data were anticipated for some variables. The number of observations included in each analysis is reported whenever applicable. No statistical imputation was performed.

### 2.12. Statistical Analysis

Statistical analyses were performed using GraphPad Prism version 10.4.2 (GraphPad Software, San Diego, CA, USA). Data distribution was assessed using the Shapiro–Wilk test. Normally distributed continuous variables are presented as mean ± standard deviation (SD), whereas non-normally distributed variables are presented as median with interquartile range (IQR) or range, as appropriate.

### 2.13. Ethical Considerations

The study was approved by the Institutional Research, Ethics, and Biosafety Committees of the National Institute of Pediatrics (Protocol number 2022/051). Written informed consent for molecular analyses was obtained from parents or legal guardians before genetic testing. All families received pre-test and post-test genetic counseling. The study was conducted in accordance with the ethical principles of the Declaration of Helsinki and applicable national regulations governing biomedical research involving human participants.

## 3. Results

### 3.1. Cohort Characteristics

A total of 88 patients from 80 unrelated families with a biochemical diagnosis of MSUD were included in the study. Seven families had two affected siblings, and one family had three affected members. The cohort comprised 46 males (52.3%) and 42 females (47.7%). Consanguinity was recorded in 19/80 families (23.8%).

Most patients presented with the classic neonatal form of the disease (80/88, 91%). Two patients were classified as intermediate (2/88, 2.2%). None of the patients had an intermittent or thiamine-responsive presentation. Clinical symptoms appeared during the neonatal period in 73/88 patients (83%), in contrast, only 9/88 patients (10.2%) presented after the neonatal period. The remaining six patients (6.8%) were properly screened and promptly treated, and they never presented with symptoms. The main clinical data affecting the neurological, gastrointestinal, and respiratory systems, as well as the patient’s biochemical characteristics, are shown in Table 1.

A total of 73/88 (83%) patients presented with acute metabolic decompensation requiring hospitalization as their initial clinical manifestation. The median age at symptom onset was 6 days (min 3 days, max 41 months). The clinical manifestations of the disease are shown in Table 1.

### 3.2. Biochemical Characteristics

**Figure 2:**
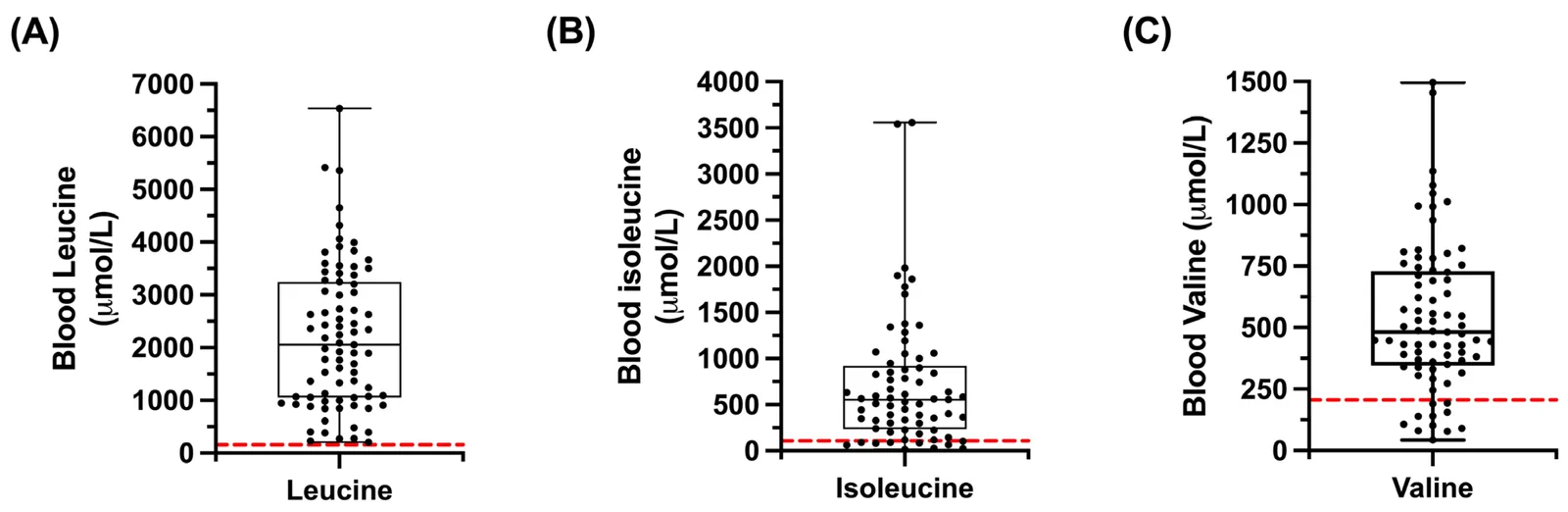
Branched-chain amino acids of Mexican maple syrup urine disease patients at the moment of diagnosis. Median with minimum and maximum values are shown. Reference values of amino acids are highlighted in red dotted lines (leucine: 155 μmol/L, isoleucine: 108 μmol/L and valine: 206 μmol/L) [29]. (**A**) Leucine; (**B**) Isoleucine and (**C**) Valine blood concentrations.

Plasma BCAA concentrations at diagnosis were available for all patients. Plasma leucine concentrations were markedly elevated in all patients (median 2074 μmol/L, min 209 μmol/L, max 6538 μmol/L), and most were accompanied by increased valine and isoleucine levels. Alloisoleucine was available in a few patients (20/88, 22.8%) evaluated after the implementation of this assay in our institutional laboratory (median 280 μmol/L, min 21 μmol/L, max 1435 μmol/L). The distribution of plasma leucine, valine, and isoleucine concentrations at diagnosis is shown in Figure 2.

### 3.3. Newborn Screening Outcomes

**Figure 3:**
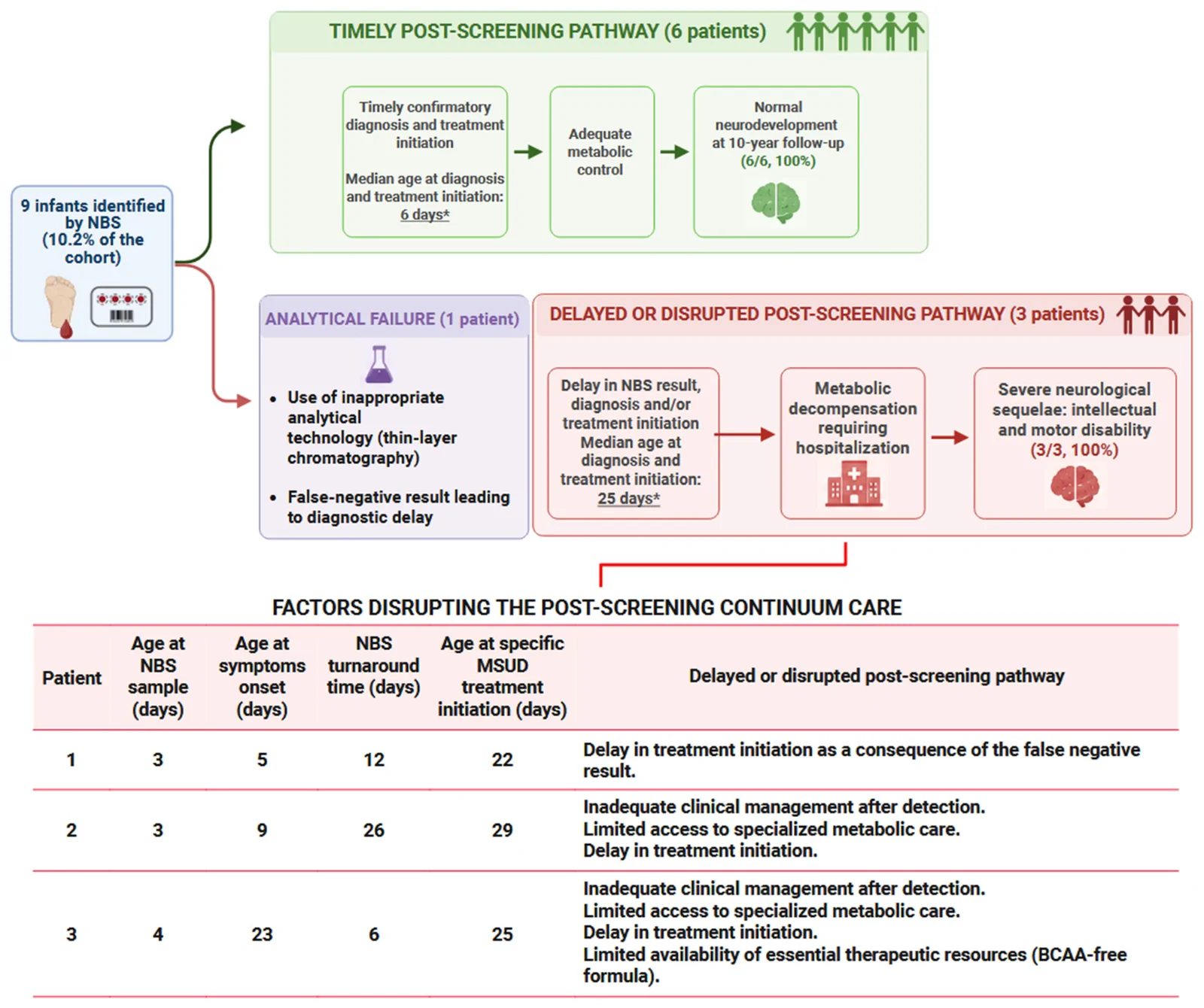
Long-term outcomes of newborn screening for MSUD according to the timeliness and continuity of post-screening metabolic care. Timely diagnosis and treatment were associated with adequate metabolic control and normal neurodevelopment, whereas analytical failure and delays or disruptions in care were associated with metabolic decompensation and severe neurological sequelae. * Median age at diagnosis and treatment initiation was statistically different between groups (*p* = 0.02).

Nine patients (10.2%) were identified through NBS programs. Six patients received a timely confirmatory diagnosis and initiation of treatment and maintained normal age-appropriate neurodevelopment during a 10-year follow-up. In contrast, three patients experienced delays in the diagnosis and therapeutic pathway that resulted in metabolic decompensation requiring hospitalization and subsequent severe neurological disability (Figure 3).

### 3.4. Clinical Outcomes and Follow-Up

At the time of study closure, patients were classified according to survival status as alive, deceased, or lost to follow-up; as a result, data on neurological outcomes were available for only 55/88 patients. The neurological outcomes ranged from normal neurodevelopment (7/55, 12.7%), mild/moderate (4/55, 5.5%), to severe (44/55, 80%) neurodevelopmental delay or intellectual and motor disability (Table 2).

The most severe outcomes were observed among patients diagnosed after metabolic decompensation and among the three severely affected patients attributable to the delayed or disrupted post-screening pathway (Figure 3). All patients with normal neurodevelopment belonged to the successful NBS group, and one with the intermediate form who was not identified through NBS.

The overall mortality in this study was 26/55 (47.2%), highlighting that none of the successfully screened patients died (Table 2), nor did those with the intermediate for of the disease.

### 3.5. MSUD Genotypic Characterization

This was available for 33 unrelated patients. A total of 31 distinct variants were identified across *BCKDHA*, *BCKDHB*, and *DBT* genes, but no pathogenic or likely pathogenic variants were identified in *DLD* (Table 3).

**Figure 4:**
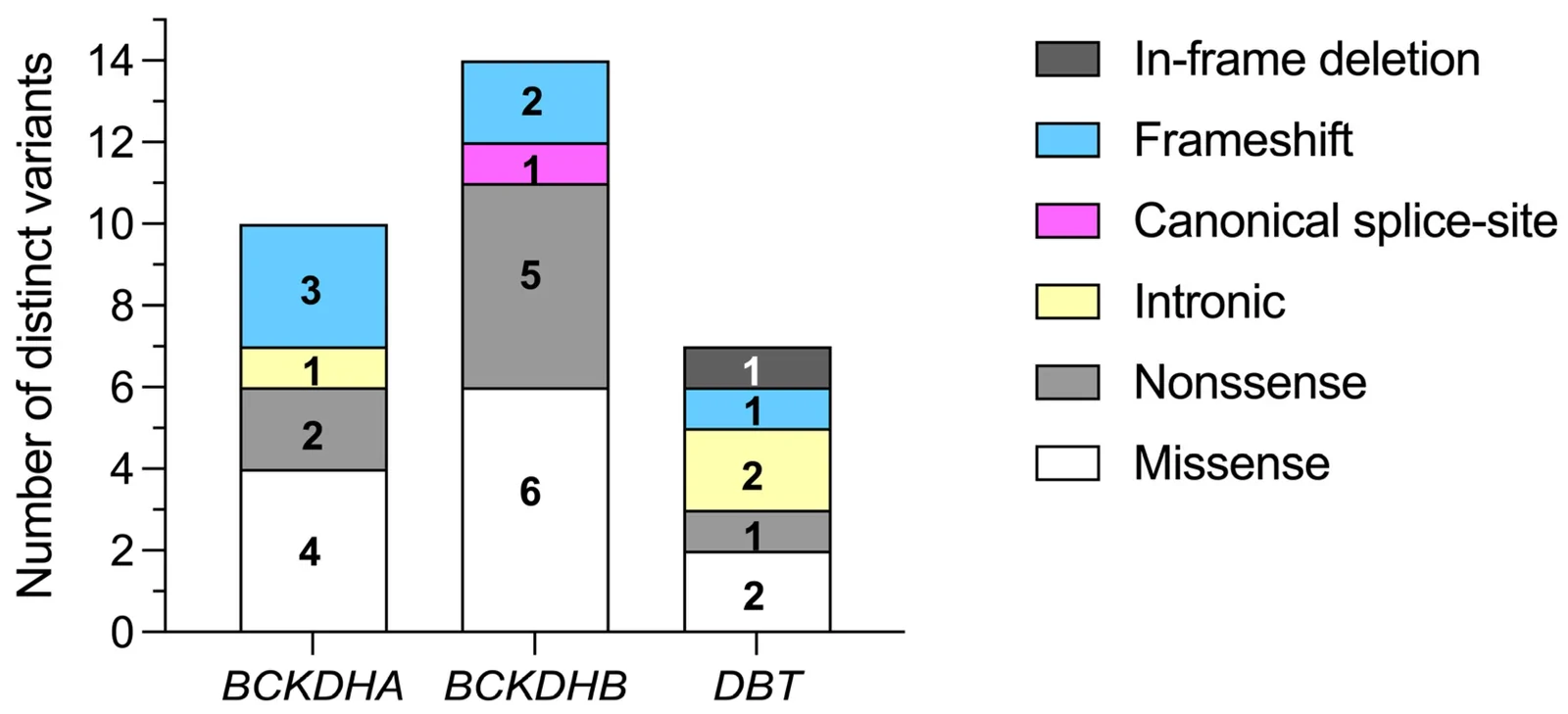
Distribution and gene variant classification regarding their predicted effect on protein from MSUD patients. The number of each variant type is depicted within the bars.

*BCKDHB* harbored the largest number of distinct variants (14/31, 45.1%), followed by *BCKDHA* (10/31, 32.2%) and *DBT* (7/31, 22.5%) (Figure 4).

The most frequent *BCKDHB* variant was c.564T>A or p.(Cys188Ter), representing 20% of all *BCKDHB* disease-associated alleles, followed by c.633+1G>T (13.3%). In *BCKDHA*, five variants each accounted for 13.3% of the identified MSUD alleles: c.691del, c.796_797delinsT, c.979G>A, c.1226T>G, and c.1267C>T. Within *DBT*, three recurrent variants (c.75_76del, c.263_265del, and c.1261G>T) were identified; each represented 20% of the disease-associated alleles (Table 3).

### 3.6. Variant Classification

Missense variants represented the most frequent variants in all genes except *DBT*, in which missense and intronic variants had the same proportion (Figure 4). Eighteen variants predicted to disrupt protein synthesis (pLOF type), represented the largest allelic proportion (18/31, 58%) (Table 3). The highest proportion of pLOF alleles was observed in *DBT* (5/7, 71.4%), followed by *BCKDHB* (8/14, 57.1%) (Table 3). Instead, missense variants without predicted LOF (pnLOF) accounted for most of the remaining variants (12/31, 38.7%). The c.109-15T>A variant, located in intron 1 of *BCKDHA* and currently classified as VUS in the dbSNP (rs1212182432) and ClinVar (2140915) databases, herein achieves the PM2, PM3, PP3, and PP4 ACMG/AMP variant pathogenicity criteria. In silico evaluation through MaxEnt, NNSplice, and GeneSplicer programs contained in the module of splicing effect predictors of the Alamut Visual Plus v.1.13 software (© 2025 SOPHiA GENETICS, Rolle, Switzerland) predicts, with a mean probability of 98.4% (91.47–99.96%) an impact on correct splicing of exon 2 due to alteration of the polypyrimidine tract and the possible activation of an intronic cryptic acceptor splicing site. Thus, it was reclassified as predicted pLOF.

### 3.7. Genotype–Phenotype Observations

Biallelic pathogenic variants were identified in 32 of 33 patients subjected to massive parallel sequencing, and only one showed a monoallelic *BCKDHA* genotype for c.117dup or p.(Arg40Glnfs*11) (MSUD002, Table 4). We identified 29 distinct genotypes distributed across *BCKDHA*, *BCKDHB*, and *DBT* among these 33 patients, demonstrating marked molecular heterogeneity within the Mexican cohort (Table 4).

In total, 16 of the 29 genotypes (55.1%) were homozygous; seven of them were identified in consanguineous families (*N* = 7/16, 43.8%), while in the compound heterozygous families, none showed documented consanguinity. No predominant genotype was observed, as nearly all genotypes were restricted to individual families and only a few were identified in two unrelated families. Most of the genotypes were composed of pLOF variants (18/29, 62%), while 20.7% consisted of two pnLOF variants and 20.7% consisted of one pLOF and one pnLOF variant. In one case (3.4%), the genotype was composed of one pnLOF variant and one pLOF variant (MSUD001 patient, Table 4).

## 4. Discussion

Our principal finding is that favorable neurological outcomes in MSUD depended not only on NBS but also on the timely delivery of specialized metabolic care after diagnosis (Table 2). Most of the cohort was characterized by a predominance of severe classic neonatal disease (Table 1), which was similar to the previously described clinical picture with a high burden of neurological disability and mortality [1] (Table 2). It is important to note that all patients who received prompt confirmatory diagnosis and immediate treatment after NBS maintained normal neurodevelopment during long-term follow-up (Figure 3). In contrast, the patients who experienced delays in diagnosis, referral, or treatment developed severe neurological sequelae despite being identified through NBS (Figure 3). These findings emphasize that, for time-critical disorders such as MSUD, NBS should be considered the entry point to a coordinated continuum of specialized metabolic care rather than an isolated diagnostic intervention.

The high burden of adverse clinical outcomes observed in this cohort likely reflects the combined influence of biological and healthcare-related factors. Most patients presented with the classic neonatal phenotype, which is associated with rapid metabolic decompensation and a high risk of irreversible neurological injury. Despite marked genotypic heterogeneity in MSUD (Figure 4), we documented a predominance of pLOF variants (Table 3), suggesting that severe disruption of BCKDH activity contributed to the high frequency of neonatal presentation. However, genetic severity alone does not fully explain the observed poor outcomes. Delays in diagnosis, limited access to specialized metabolic care, and barriers to timely treatment probably amplified the neurological consequences of early metabolic decompensation. Our findings do not demonstrate that variant class independently determines neurological outcome. Rather, the striking contrast between promptly treated and delayed-treatment patients suggests that the duration of metabolic intoxication before effective therapy is a major determinant of neurological outcome, superimposed on the underlying biochemical phenotype. Similar challenges have been reported in other middle-income countries, including the Philippines, where mortality and long-term neurological disability remain strongly influenced by healthcare access in addition to disease severity [30].

The observed genetic heterogeneity of MSUD in our study population is consistent with the molecular architecture of MSUD worldwide, in which more than 300 pathogenic variants have been reported and most outbred populations lack a single predominant disease-causing variant [1].

In 19 of the 80 studied families, consanguinity was documented (23.8%). This proportion falls within the broad range of consanguinity reported in MSUD cohorts worldwide of approximately 23–88% [31].

A monoallelic *BCKDHA* genotype was found in one of the 33 analyzed patients (3%) (Patient MSUD002, Table 4). Strauss et al., in the MSUD patients from the Clinic for Special Children Laboratory, found a similar proportion of monoallelic genotypes (2.8%); however, in contrast to our findings, those authors identified all monoallelic genotypes in the *DBT* gene [1]. This monoallelic genotype could be due to pathogenic deep intronic variants that were not identified by WES. These type of intronic variants have already been previously reported in *BCKDH* gene [4]. Further studies such as long-read whole-genome sequencing or RNA-seq methodologies are needed to discard this hypothesis in our patient.

The distribution of variants among genes was also comparable to that reported in other non-founder populations. *BCKDHB* was the most frequently affected gene in our cohort, followed by *BCKDHA* and *DBT*, closely resembling observations from Brazil, Chile, and China, where *BCKDHB* consistently accounts for the largest proportion of genetic diagnoses with 52.6%, 88%, and 50%, respectively [15,16,32]. In contrast, our findings differ from founder populations such as the Old Order Mennonites of Pennsylvania, where a single *BCKDHA* variant predominates, as well as from Portuguese and Philippine cohorts, in which *BCKDHA* and *DBT*, respectively, represent the major disease-associated genes [1,4,5].

Consistent with these observations, no predominant pathogenic variant was identified in the Mexican cohort. Although the *BCKDHB* c.564T>A or p.(Cys188*) variant was the most frequent allele (accounting for 20% of all *BCKDHB* alleles), most variants were observed only once or twice and were restricted to individual families. This pattern closely resembles that reported in Spain, where extensive allelic heterogeneity (36 distinct variants identified among 33 patients) and the absence of a major founder mutation have also been described [33]. Together, these findings support the existence of multiple private mutations in the Mexican population rather than a founder effect.

pLOF/pLOF represented most of the disease-associated genotypes in our cohort (62%) (Table 4), supporting the predominance of the severe classic neonatal phenotype; however, genotypes composed of two pnLOF variants (20.7%) were also present in this classical form of the disease. Because complete disruption of the BCKDH complex is expected to severely reduce or abolish residual enzymatic activity, this molecular profile probably contributed to the high frequency of neonatal presentation observed in our patients. Nevertheless, genotype alone does not fully explain clinical severity.

Several biological and healthcare-related factors probably explain the limited genotype–phenotype correlations observed in MSUD. First, the timing of diagnosis and treatment initiation profoundly modifies disease expression because early metabolic intervention limits the duration of leucine neurotoxicity and may attenuate the severity associated with a given genotype [10,15,32,33]. This fact can be exemplified by the patient MSUD025 (Table 4), who was detected late and presented a severe phenotype, but in contrast, her sibling, who was timely screened and treated, is currently asymptomatic. Furthermore, other factors such as the type of variant, interallelic complementation, residual BCKDH activity, protein folding, stability, oligomerization, or catalytic efficiency could also influence the phenotype [10]. Finally, long-term outcomes are further influenced by access to specialized metabolic care, dietary adherence, and the frequency of metabolic decompensations. Our findings therefore suggest that genetic background establishes the biological severity of MSUD, whereas timely diagnosis and access to specialized metabolic care substantially modify its clinical expression. The patient MSUD029 harboring the *DBT* c.670G>T or p.(Glu224*) variant illustrates this concept. Although this variant has recently been associated with thiamine responsiveness when paired with the missense allele c.916T>C or p.(Ser306Pro) [34], our patient carried a second pLOF variant and presented with classic neonatal MSUD, severe neurological impairment, and no apparent response to thiamine supplementation. Although this observation is based on a single patient, it suggests that therapeutic responsiveness may depend on the whole biallelic effect and the level of residual BCKDH activity rather than on a single variant alone.

Collectively, our findings expand the molecular spectrum of MSUD in Mexico and support the use of comprehensive sequencing approaches, such as targeted next-generation sequencing panels or clinical exome sequencing, as first-line molecular diagnostic strategies in populations without founder mutations. These approaches not only improve diagnostic yield but also provide the molecular basis for accurate genetic counseling and family cascade testing [35].

The experiences of the nine patients diagnosed through NBS provide the clearest illustration of this study’s key message. Six of the nine patients (66.6%) received prompt confirmatory diagnosis and immediate initiation of treatment, and all maintained normal neurodevelopment during more than 10 years of follow-up (Figure 3). In contrast, the remaining three patients developed severe neurological sequelae despite being identified through NBS because critical failures occurred after screening, including false-negative analytical results, delayed referral and treatment initiation, limited metabolic management expertise, or limited access to specialized medical formulas. These observations demonstrate that in MSUD, particularly in resource-limited settings, neurological outcome depended less on the availability of NBS itself than on the success of the post-analytical NBS phase following a positive screening result (Figure 3). Our findings are consistent with emerging evidence indicating that the success of NBS extends beyond analytical performance. In a recent meta-analysis including 1141 patients with MSUD, Scharre et al. reported substantially lower mortality (3%) among patients identified through NBS than among those diagnosed after symptom onset (14.6%); however, favorable outcomes were also strongly associated with healthcare quality indicators and timely access to specialized metabolic care [10]. Likewise, Servais emphasized that prognosis depends not only on early diagnosis but also on rapid confirmatory testing, frequent biochemical monitoring, specialized nutritional support, timely access to valine and isoleucine supplementation, and availability of dialysis when indicated [36]. Together, these studies support the concept that NBS should be viewed as the entry point to an integrated continuum of metabolic care rather than as an isolated diagnostic intervention.

These findings are particularly relevant because MSUD is one of the most time-critical disorders included in NBS programs. In infants with the classic phenotype, metabolic intoxication may begin within the first days of life, often before conventional screening results become available. Consequently, delays at any point between specimen collection, confirmatory diagnosis, referral, and treatment initiation may substantially reduce the preventive potential of NBS [7,37].

The high mortality and neurological disability observed in our cohort likely reflect not only the predominance of severe classic MSUD but also inequities in access to specialized metabolic care. Similar observations have been reported in the Philippines, where delays in diagnosis, limited availability of metabolic specialists, inadequate access to medical foods, and recurrent metabolic crises substantially influenced survival and long-term neurological outcomes [30,38]. Although the frequency of recurrent metabolic crises was not systematically evaluated in our cohort, repeated episodes of metabolic instability probably contributed to the neurological burden observed among survivors. These findings underscore the importance of strengthening specialized metabolic services alongside the expansion of NBS programs.

### Study Limitations

This study has several limitations. First, its retrospective design and the long inclusion period (1995–2026) encompass substantial changes in NBS availability, biochemical and molecular diagnostic methods, and clinical management, which may have introduced temporal heterogeneity into the cohort. Second, as a national tertiary referral center for inherited metabolic disorders, our institution is more likely to receive patients with severe clinical presentations or delayed diagnoses, potentially overestimating the frequency of neurological disability and mortality. Third, complete molecular characterization and long-term neurological follow-up were unavailable for all patients, limiting the achievement of a more representative MSUD genotypic spectrum in Mexico, as well as a more comprehensive genotype–phenotype and outcome comparisons.

Nevertheless, these limitations should be interpreted in the context of the study’s strengths. This represents the largest MSUD cohort reported from Mexico, with more than three decades of clinical experience and comprehensive integration of biochemical, molecular, and long-term clinical data. Moreover, the inclusion of patients managed under different diagnostic and healthcare conditions provided a unique opportunity to evaluate how access to NBS and specialized metabolic care influenced clinical outcomes over time.

## 5. Conclusions

This study provides the largest clinical and genotypic characterization of MSUD reported in Mexico and demonstrates that favorable neurological outcomes depend not only on early identification through NBS but also on the timely delivery of specialized metabolic care. Although the predominance of severe pLOF/pLOF genotypes probably contributed to the high frequency of classic neonatal disease (62%), healthcare-related factors substantially modified clinical outcomes.

Our findings reinforce the concept that, for time-critical disorders such as MSUD, NBS needs to move beyond expanding screening programs alone, and it should be regarded as the first step in an integrated continuum of care rather than as an isolated diagnostic intervention. National NBS strategies should prioritize the inclusion of this time critical disorder and the shortening of the interval between a positive screening result and treatment initiation, ensuring uninterrupted access to specialized metabolic formulas, implementing standardized emergency management protocols, improving regional access to expert metabolic centers, and strengthening training for healthcare professionals in inherited metabolic disorders. In this context, NBS should be viewed not as an isolated public health intervention but as the first in a coordinated system of specialized metabolic care, which may substantially improve survival and long-term neurodevelopmental outcomes in Mexican MSUD patients and others from middle-income countries.

## Figures and Tables

**Table 1:** Percentage of Age-Related Clinical Manifestations in Mexican MSUD Patients (*N* = 88 *).

		Classic Form			Intermediate Form **
		1st week (*N* = 50)	1st month (*N* = 23)	Early infancy (*N* = 7)	Infancy (*N* = 2)
Neurological	Irritability	52%	61%	86%	50%
	Opisthotonus	46%	35%	29%	100%
	Seizures	34%	39%	29%	100%
	Hypotonia	30%	39%	14%	50%
	Progressive neurologic deterioration	30%	35%	29%	0%
	Letargy	12%	48%	29%	50%
	Coma	4%	9%	0%	50%
	Dystonia	2%	9%	0%	0%
Systemic	Sweet odor	82%	87%	100%	100%
	Metabolic acidosis	62%	83%	57%	50%
	Increased anion gap	60%	78%	57%	0%
	Ketosis	28%	52%	14%	50%
	Hyperammonemia	24%	35%	0%	0%
	Ketonuria	22%	43%	57%	50%
	Hypoglycemia	20%	22%	0%	50%
	Anemia	12%	9%	14%	50%
	Trombocitopenia	2%	0%	14%	0%
	Hyperglycemia	2%	0%	0%	0%
	Leukopenia	2%	0%	0%	0%
Gastrointestinal	Feeding difficulties	52%	43%	29%	100%
	Dehydration	32%	61%	14%	50%
	Vomiting	26%	39%	14%	50%
	Diarrhea	16%	9%	0%	100%
	Constipation	2%	0%	14%	0%
Respiratory	Apnea	18%	17%	29%	50%
	Dysnea	8%	9%	0%	0%
	Tachypnea	4%	22%	14%	50%

* Including 6 asymptomatic NBS patients. ** These patients were not detected by NBS.

**Table 2:** Neurological outcome and mortality in Mexican MSUD patients at the last follow-up visit evaluation (*N* = 55 *).

Outcome	Unscreened		Screened			
	Number of Patients	Mortality	Successful NBS	Mortality	Unsuccessful NBS	Mortality
Deceased <6-years old with profound neurodevelopmental delay	16/55 (29%)	16/55 (29%)	0	0	0	1/55 (1.8%)
Deceased >6-years old with profound intellectual disability	9/55 (16.4%)	9/55 (16.4%)	0	0	0	0
Alive <6-years old with global neurodevelopmental delay	3/55 (5.5%)	0	0	0	1/55 (1.8%)	0
Alive >6-years old with profound intellectual disability	14/55 (25.5%)	0	0	0	1/55 (1.8%)	0
Alive >6-years old with mild/moderate intellectual disability	3/55 (5.5%)	0	0	0	0	0
Alive with normal neurological development	1/55 (1.8%)	0	6/55 (10.9%)	0	0	0
TOTAL	46/55 (83.6%)	25/55 (45.4%)	6/55 (10.9%)	0	2/55 (3.6%)	1/55 (1.8%)

* The remaining 33 patients (37%) were lost to follow-up.

**Table 3:** Variants located in *BCKDHA*, *BCKDHB* and *DBT* in the Mexican patients with MSUD.

Variant	c.DNA ClinVar and dbSNP Entries	Protein Change	Predicted Functional Classification Allele	Exon/ Intron	Number of Alleles (Frequency %)	ACMG Classification
***BCKDHA*** **(NM_000709.4) *N* = 15 Alleles**						
1	c.691del VCV004814471.2	p.(Val231Serfs*99)	pLOF	6	2 (13.3)	Pathogenic (PVS1, PM2, PM3, PP5)
2	c.796_797delinsT VCV002782918.3 rs754303047	p.(Asn266Thrfs*64)	pLOF	6	2 (13.3)	Pathogenic (PVS1, PP5, PM2)
3	c.979G>A VCV000093385.23 rs398123515	p.(Glu327Lys)	pnLOF	7	2 (13.3)	Pathogenic (PP2, PP3, PP5, PM2)
4	c.1226T>G VCV000002378.11 rs137852872	p.(Phe409Cys)	pnLOF	9	2 (13.3)	Pathogenic (PP5, PM5, PM2, PP2, PP3, PM3)
5	c.1267C>T	p.(Gln423*)	pLOF	9	2 (13.3)	Pathogenic (PVS1, PM2, PM3)
6	c.109-15T>A VCV002140915.1 rs1212182432	p.(?)	pLOF	1	1 (6.7)	Likely pathogenic (PM2, PM3, PP3, PP4)
7	c.117dup VCV000166741.26 rs398123489	p.(Arg40Glnfs*11)	pLOF	2	1 (6.7)	Pathogenic (PVS1, PP5, PM2)
8	c.632C>T VCV000093365.23 rs398123503	p.(Thr211Met)	pnLOF	5	1 (6.7)	Pathogenic (PM2, PM3, PP2, PP3, PP5, PS3)
9	c.890G>A VCV000093376.22 rs200137189	p.(Arg297His)	pnLOF	7	1 (6.7)	Pathogenic (PP5, PP3, PM1, PM2, PP2, PM3, PM5)
10	c.1192G>T VCV000553717.3 rs201168715	p.(Glu398*)	pLOF	9	1 (6.7)	Pathogenic (PVS1, PP5, PM2, PM3)
***BCKDHB*** **(NM_183050.4) *N* = 30 Alleles**						
1	c.564T>A VCV000580585.14 rs774306610	p.(Cys188*)	pLOF	5	6 (20)	Pathogenic (PVS1, PP5, PM2)
2	c.633+1G>T VCV000553536.9 rs398124589	p.(?)	pLOF	5	4 (13.3)	Pathogenic (PVS1, PP5, PM2, PM3, PS3)
3	c.667G>C VCV003775409.1	p.(Gly223Arg)	pnLOF	6	3 (10)	Likely Pathogenic (PP2, PP3, PM2, PM3)
4	c.970C>T VCV000096621.29 rs398124603	p.(Arg324*)	pLOF	9	3 (10)	Pathogenic (PVS1, PP5, PM2, PM3, PS3)
5	c.1087T>A VCV000096568.18 rs398124565	p.(Tyr363Asn)	pnLOF	10	3 (10)	Pathogenic (PM1, PM2, PM3, PP2, PP3, PP5, PS4)
6	c.152del VCV000527129.10 rs867612284	p.(Val51Glyfs*21)	pLOF	1	2 (6.8)	Pathogenic (PVS1, PP5, PM2)
7	c.348del VCV000457148.16 rs1400121541	p.(Asp117Ilefs*113)	pLOF	4	2 (6.8)	Pathogenic (PVS1, PP5, PM2)
8	c.487G>T VCV000370827.19 rs1057516799	p.(Glu163*)	pLOF	5	1 (3.3)	Pathogenic (PVS1, PP5, PM2, PM3)
9	c.506A>G VCV000096587.22 rs398124580	p.(Tyr169Cys)	pnLOF	5	1 (3.3)	Likely Pathogenic (PM1, PM2, PM3, PP2, PP3, PP4, PP5)
10	c.509G>C VCV000096592.13 rs371518124	p.(Arg170Pro)	pnLOF	5	1 (3.3)	Likely Pathogenic (PM1, PM2, PM5, PP2, PP3, PP5)
11	c.713T>C VCV004294017.1	p.(Phe238Ser)	pnLOF	6	1 (3.3)	Likely Pathogenic (PM2, PM3, PP2, PP3, PP4)
12	c.838C>T VCV004817028.1	p.(Gln270*)	pLOF	7	1 (3.3)	Pathogenic (PVS1, PM2, PM3, PP4, PP5)
13	c.853C>T VCV000096615.47 rs398124598	p.(Arg285*)	pLOF	8	1 (3.3)	Pathogenic (PVS1, PM2, PP5)
14	c.991G>A VCV001691427.11 rs1777825845	p.(Ala331Thr)	pnLOF	9	1 (3.3)	Pathogenic (PM1, PM2, PM3, PP2, PP3, PP4, PP5)
***DBT*** **(NM_001918.5) *N* = 20 Alleles**						
1	c.75_76del VCV000011950.37 rs768832921	p.(Cys26Trpfs*2)	pLOF	2	4 (20)	Pathogenic (PVS1, PP5, PM2, PM3)
2	c.263_265del VCV000527136.13 rs1217050849	p.(Glu88del)	pLOF	4	4 (20)	Pathogenic (PM1, PM2, PM3, PM4, PS4, PP5)
3	c.1261G>T VCV000203669.7 rs796051945	p.(Gly421Trp)	pnLOF	10	4 (20)	Likely Pathogenic (PM2, PP4, PS4)
4	c.434-15_434-4del VCV000166983.20 rs727503895	p.(?)	pLOF	4	3 (15)	Pathogenic (PP4, PP5, PM2, PM3, BP4, PS3)
5	c.1210-3T>A VCV001906704.4 rs758470589	p.(?)	pLOF	9	3 (15)	Pathogenic (PM2, PM3, PP3, PP4, PS4)
6	c.242T>G	p.(Val81Gly)	pnLOF	3	1 (5)	Likely Pathogenic (PM1, PM2, PM3, PP2, PP3, PP4)
7	c.670G>T VCV000094009.34 rs74103423	p.(Glu224*)	pLOF	6	1 (5)	Pathogenic (PVS1, PM2, PM3, PP5)

pLOF = Predicted loss of function; pnLOF = Predicted no loss of function.

**Table 4:** Genotypes Of Patients With MSUD.

Patient ID	Genotype (cDNA)	Genotype (Protein)	Predicted Functional Classification	Number of Patients	Observed Phenotype
***BCKDHA*** **(NM_000709.4) *N* = 8**					
MSUD001	c.[109-15T>A];[979G>A]	p.[?];[Glu327Lys]	pLOF/pnLOF	1	Classic
MSUD002	c.[117dup];[=]	p.[Arg40Glnfs*11];[=]	pLOF/=	1	Classic
MSUD003	c.[632C>T];[979G>A]	p.[Thr211Met];[Glu327Lys]	pnLOF/pnLOF	1	Classic
MSUD004	c.[691del];[691del]	p.[Val231Serfs*99];[Val231Serfs*99]	pLOF/pLOF	1	Classic
MSUD059	c.[796_797delinsT];[796_797delinsT]	p.[Asn266Thrfs*64];[Asn266Thrfs*64]	pLOF/pLOF	1	Classic
MSUD005	c.[890G>A];[1192G>T]	p.[Arg297His];[Glu398*]	pnLOF/pLOF	1	Asymptomatic
MSUD006	c.[1226T>G];[1226T>G]	p.[Phe409Cys];[Phe409Cys]	pnLOF/pnLOF	1	Classic
MSUD007 ✦	c.[1267C>T];[1267C>T]	p.[Gln423*];[Gln423*]	pLOF/pLOF	1	Classic
***BCKDHB*** **(NM_183050.4) *N* = 14**					
MSUD013 MSUD014	c.[564T>A];[564T>A]	p.[Cys188*];[Cys188*]	pLOF/pLOF	2	Classic
MSUD008	c.[152del];[152del]	p.[Val51Glyfs*21];[Val51Glyfs*21]	pLOF/pLOF	1	Classic
MSUD009	c.[348del];[348del]	p.[Asp117Ilefs*113];[Asp117Ilefs*113]	pLOF/pLOF	1	Classic
MSUD010	c.[487G>T];[633+1G>T]	p.[Glu163*];[p.?]	pLOF/pLOF	1	Classic
MSUD011	c.[506A>G];[838C>T]	p.[Tyr169Cys];[Gln270*]	pnLOF/pLOF	1	Classic
MSUD012	c.[509G>C];[713T>C]	p.[Arg170Pro];[Phe238Ser]	pnLOF/pnLOF	1	Classic
MSUD015	c.[564T>A];[970C>T]	p.[Cys188*];[Arg324*]	pLOF/pLOF	1	Classic
MSUD016	c.[564T>A];[1087T>A]	p.[Cys188*];[Tyr363Asn]	pLOF/pnLOF	1	Classic
MSUD017	c.[633+1G>T];[633+1G>T]	p.[?];[?]	pLOF/pLOF	1	Classic
MSUD018	c.[633+1G>T];[991G>A]	p.[?];[Ala331Thr]	pLOF/pnLOF	1	Classic
MSUD019	c.[667G>C];[667G>C]	p.[Gly223Arg];[Gly223Arg]	pnLOF/pnLOF	1	Classic
MSUD020	c.[667G>C];[853C>T]	p.[Gly223Arg];[Arg285*]	pnLOF/pLOF	1	Classic
MSUD021	c.[970C>T];[970C>T]	p.[Arg324*];[Arg324*]	pLOF/pLOF	1	Classic
MSUD022	c.[1087T>A];[1087T>A]	p.[Tyr363Asn];[Tyr363Asn]	pnLOF/pnLOF	1	Classic
***DBT*** **(NM_001918.5) *N* = 7**					
MSUD023 MSUD024	c.[75_76del];[75_76del]	p.[Cys26Trpfs*2];[Cys26Trpfs*2]	pLOF/pLOF	2	Classic
MSUD026 MSUD027	c.[263_265del];[263_265del]	p.[Glu88del];[Glu88del]	pLOF/pLOF	2	Classic
MSUD031 MSUD032	c.[1261G>T];[1261G>T]	p.[Gly421Trp ];[Gly421Trp]	pnLOF/pnLOF	2	Classic
MSUD025	c.[242T>G];[1210-3T>A]	p.[Val81Gly];[?]	pnLOF/pLOF	1	Asymptomatic
MSUD028	c.[434-15_434-4del];[434-15_434-4del]	p.[?];[?]	pLOF/pLOF	1	Classic
MSUD029	c.[434-15_434-4del];[670G>T]	p.[?];[Glu224*]	pLOF/pLOF	1	Classic
MSUD030	c.[1210-3T>A];[1210-3T>A]	p.[?];[?]	pLOF/pLOF	1	Classic

Those patients with NBS are shaded in gray. The NBS pathway of patients MSUD003 and MSUD007 was unsuccessful; while those of patients MSUD005 and MSUD025 was successful. pLOF: predicted loss of function. ✦ The genotypic and phenotypic information of this patient was uploaded to LOVD with ID accesses: variant LOVD ID: #0001083205; Individual LOVD ID: #00483770.

## Data Availability

The original contributions presented in the study are included in the article; further inquiries can be directed to the corresponding author.

## Abbreviations

The following abbreviations are used in this manuscript:

MSUD	Maple syrup urine disease
BCKDH	Branched-chain α-ketoacid dehydrogenase complex
BCAAs	Branched-chain amino acids
BCKDHA	Branched-chain keto acid dehydrogenase E1α subunit
BCKDHB	Branched-chain keto acid dehydrogenase E1β subunit
DBT	Dihydrolipoamide branched-chain transacylase
DLD	Dihydrolipoamide dehydrogenase
pLOF	Predicted Loss-of-function variant
pnLOF	Predicted non-loss-of-function variant
VUS	Variant of uncertain significance
